# Two Cases of Sitosterolemia Falsely Diagnosed as Familial Hypercholesterolemia: Could Digging Deeper Have Avoided Harm?

**DOI:** 10.1210/jcemcr/luae086

**Published:** 2024-05-03

**Authors:** Simon-Pierre Guay, Martine Paquette, Chantal Blais, Géraldine Gosse, Alexis Baass

**Affiliations:** Genetic Dyslipidemias Clinic of the Montreal Clinical Research Institute, Montréal, Québec H2W 1R7, Canada; Department of Medicine, Division of Endocrinology, Université de Montréal, Montréal, Québec H3T 1J4, Canada; Department of Pediatrics, Division of Medical Genetics, Université de Sherbrooke, Sherbrooke, Québec J1H 5N4, Canada; Genetic Dyslipidemias Clinic of the Montreal Clinical Research Institute, Montréal, Québec H2W 1R7, Canada; Genetic Dyslipidemias Clinic of the Montreal Clinical Research Institute, Montréal, Québec H2W 1R7, Canada; Genetic Dyslipidemias Clinic of the Montreal Clinical Research Institute, Montréal, Québec H2W 1R7, Canada; Genetic Dyslipidemias Clinic of the Montreal Clinical Research Institute, Montréal, Québec H2W 1R7, Canada; Department of Medicine, Divisions of Experimental Medicine and Medical Biochemistry, McGill University, Montréal, Québec H3A 0G4, Canada

**Keywords:** sitosterolemia, *ABCG5*, phytosterol, macrothrombocytopenia, ezetimibe

## Abstract

Sitosterolemia is a rare monogenic lipid disease characterized by the excessive uptake of phytosterols and their accumulation in blood and tissues. Clinically, it can present with hypercholesterolemia and xanthomas, often causing it to be misdiagnosed as familial hypercholesterolemia (FH). The diagnosis of sitosterolemia can easily be confirmed and distinguished from FH with a sterol profile and genetic investigations. Here, we report a sibship of 2 sisters with sitosterolemia initially misdiagnosed as FH. This case report illustrates the importance of considering rare conditions, such as sitosterolemia, as a differential diagnosis in patients with hypercholesterolemia, xanthomas, and hematologic anomalies. It also emphasizes the underdiagnosis of sitosterolemia and the benefits of using sterol profiles and genetic testing in the diagnostic process to initiate the appropriate therapy and avoid harm to patients.

## Introduction

Sitosterolemia (OMIM: 618666 and 210250) is a rare autosomal recessive disorder of noncholesterol sterol metabolism. It is caused by biallelic pathogenic variants in ATP-binding cassette subfamily G members 5 (*ABCG5*) or member 8 (*ABCG8*) genes involved in the excretion of sterols. Sterols are absorbed from the gastrointestinal tract via the sterol influx transporter, Nieman pick C1 like 1 (NPCL1) (1). Once in the enterocytes, unesterified cholesterol and plant sterols are mostly pumped back to the intestinal lumen via ABCG5/8 transporter. Plant sterols that are not returned to the gastrointestinal tract may reach the liver but will ultimately be excreted through the bile by the action of the hepatic ABCG5/8 transporter (1).

Plant sterols are often consumed in nuts, seeds, legumes, and vegetable oils. Usually, less than 5% of dietary plant sterols are absorbed (2). However, in individuals with sitosterolemia, there is a hyperabsorption and decreased biliary excretion of noncholesterol sterols which is associated with a high level of β-sitosterol and campesterol in plasma (usually 10-100 times higher than in normal individuals) (2). This accumulation of noncholesterol sterols in plasma and tissues is associated with premature cardiovascular disease (CVD) and xanthomas (3, 4). For this reason, sitosterolemia is often misdiagnosed as familial hypercholesterolemia (FH).

Severe hypercholesterolemia and tendinous xanthomas are widely said to be pathognomonic of FH (5, 6). However, other rare genetic dyslipidemias such as sitosterolemia can also present with a similar phenotype (7). The phenotype of sitosterolemia is broader than FH and includes abnormal liver function tests, arthralgia, splenomegaly, and hematologic findings (such as hemolytic anemia, abnormally shaped erythrocytes and macrothrombocytopenia) (3, 4). Sitosterolemia can be confirmed and distinguished from FH with a sterol profile and genetic investigations. Here, we report a sibship of 2 sisters with sitosterolemia who were initially misdiagnosed with FH.

## Case Presentation

### Case 1

**Figure 1.**
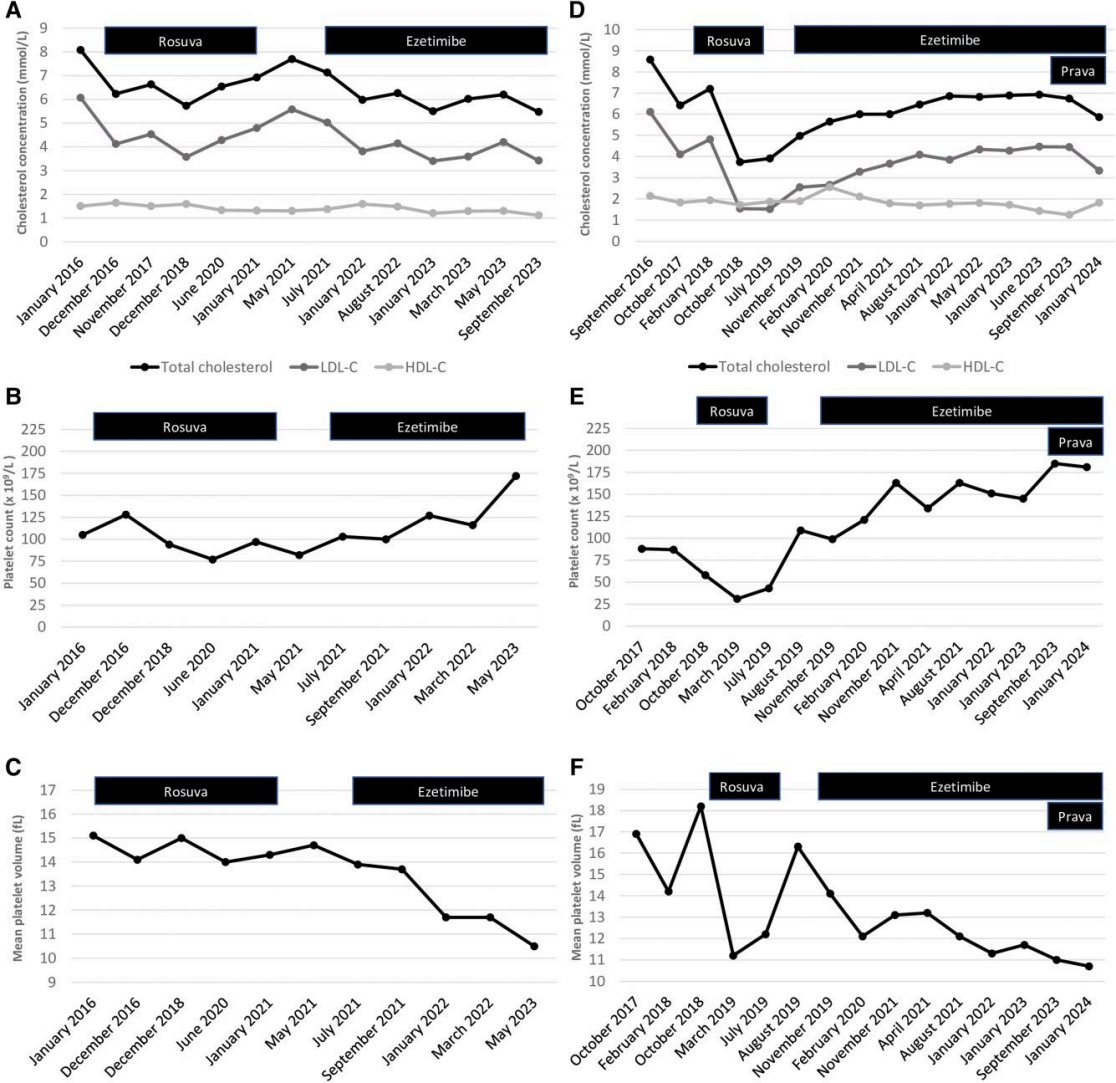
Cholesterol concentration, platelet count, and mean platelet volume according to treatment. This figure shows the cholesterol concentrations, platelet count, and mean platelet volume over time according to treatment in patient 1 (panels A-C) and patient 2 (panels D-F). HDL-C, high-density lipoprotein cholesterol; LDL-C, low-density lipoprotein cholesterol; Prava, pravastatin; Rosuva, rosuvastatin.

Patient 1 is a 58-year-old French-Canadian woman referred to the Genetic Dyslipidemias Clinic of the Montreal Clinical Research Institute in 2023. She presented with a 20-year history of hypercholesterolemia associated with xanthelasmas (Fig. 1A). She was treated with statins from 2012 to 2021, but the statins often had to be stopped following increases in liver enzymes. Since 2021, she had been treated with ezetimibe in monotherapy. She was also known for macrothrombocytopenia (platelet counts as low as 77 × 10^9^/L with mean platelet volume [MPV] up to 15 fL) (Fig. 1B and 1C), moderate hepatic steatosis, hypothyroidism, bipolar disorder, and premature coronary artery disease. She had symptoms of dyspnea, chest pain with mild facial edema, and discomfort that had worsened following the initiation of a Mediterranean diet. A coronary angiography performed at age 55 years in the context of atypical chest pain showed non–flow-limiting stenosis (<30%) of the ostium of the left main coronary artery and of the second diagonal coronary artery. The first diagonal coronary artery also had significant stenosis (70%-90%). In our clinic, she presented with a few xanthelasmas, but the physical examination was otherwise unremarkable.

### Case 2

Patient 1's sister is 59-year-old woman (patient 2) and was also referred to our clinic in 2023. Similarly to her sister, she was known for hypercholesterolemia (Fig. 1D), xanthelasma, hepatic steatosis, hypothyroidism, and bipolar disorder. Treatment with statins was initiated in 2018 but stopped in 2019 following an increase in liver enzymes. A liver biopsy showed focal necrosis most likely secondary to medication toxicity. Since 2019, she has been treated only with ezetimibe. Interestingly, she was also known for macrothrombocytopenia (platelet count 145 × 10^9^/L with MVP of 11.1 fL) (Fig. 1E and 1F). She did not report any CV symptoms but had an abnormal coronary calcium score (165.3 AU; 93rd percentile for age, sex, and race) in 2023. In our clinic, a left Achilles tendon xanthoma was noted on physical examination. The sisters do not have other siblings. Their mother was known to have hypothyroidism, hypercholesterolemia, and died of ovarian cancer. Their father is known to have hypercholesterolemia, diagnosed in midadulthood. Their maternal grandfather died of a myocardial infarction in his 60s. Patient 2 has 2 healthy children.

## Diagnostic Assessment

Considering the family history of hypercholesterolemia and CVD in each generation suggestive of an autosomal dominant inheritance, a diagnosis of FH was highly suspected for both patients. However, both were negative for the most common French-Canadian variants in the *LDLR* gene in 2021. A multigene panel for hypercholesterolemia, including *LDLR*, *APOB*, *PCSK9*, and *LDLRAP1* was negative for patient 1. However, patient 2 was found to be heterozygous for 2 variants of uncertain significance (VUS) in *APOB* (c.7565G > A; p.Arg2522Gln) and in *LDLR* (c.1085A > C; p.Asp362Ala) (8). Despite the inconclusive genetic testing, a diagnosis of probable FH was made prior to their referral to our clinic (9).

Considering the presence of xanthomas in the elder sister and the history of macrothrombocytopenia, a diagnosis of sitosterolemia was suspected. The targeted gene panel for sitosterolemia done in 2023 identified that both patients were heterozygous for 2 nonsense pathogenic variants in *ABCG5* (c.727C > T, p.Arg243* and c.1336C > T, p.Arg446*). These variants have previously been reported in individuals with sitosterolemia (10-15). Following the genetic results, a sterol profile was conducted for both patients. Although they were treated with ezetimibe, a significantly high level of β-sitosterol and campesterol was observed in both patients, confirming the diagnosis of sitosterolemia (Table 1).

**Table 1.** Sterol profiles following sitosterolemia diagnosis

		Patient 1				Patient 2		
		August 2022	March 2023	Sept 2023	Dec 2023	March 2023	Sept 2023	Dec 2023
**Treatment**	—	Ezetimibe	Ezetimibe diet	Ezetimibe diet	Ezetimibe diet	Ezetimibe	Ezetimibe diet	Ezetimibe diet Prava
**Sterol profile**								
7-Dehydrocholesterol	<0.5 µM (<20 µg/dL)	**1.9 µM (76 µg/dL)**	0 µM (0 µg/dL)	0 µM (0 µg/dL)	0 µM (0 µg/dL)	0 µM (0 µg/dL)	0 µM (0 µg/dL)	0 µM (0 µg/dL)
Cholestanol	2.5-15.4 µM (100-615 µg/dL)	**42.2 µM (1688 µg/dL)**	**49.7 µM (1988µg/dL)**	**56.0 µM (2240 µg/dL)**	**46.2 µM (1848µg/dL)**	**36.5 µM (1460 µg/dL)**	**47.9 µM (1916µg/dL)**	**28.4 µM (1136 µg/dL)**
β-Sitosterol	<24 µM (<960 µg/dL)	**484.1 µM (19 364 µg/dL)**	**350.4 µM (14 016 µg/dL)**	**361.8 µM (14 472 µg/dL)**	**395.9 µM (15 836 µg/dL)**	**318.8 µM (12 752 µg/dL)**	**402.9 µM (16 116 µg/dL)**	**247.5 µM (9900 µg/dL)**
Campesterol	<24 µM (<960 µg/dL)	**197.5 µM (7900 µg/dL)**	**146.3 µM (5852 µg/dL)**	**153.5 µM (6140 µg/dL)**	**141.0 µM (5640 µg/dL)**	**123.9 µM (4956 µg/dL)**	**174.4 µM (6976 µg/dL)**	**85.8 µM (3432 µg/dL)**
Desmosterol	<6.5 µM (<260 µg/dL)	2.3 µM (92 µg/dL)	1.8 µM (72 µg/dL)	1.6 µM (64 µg/dL)	1.4 µM (56 µg/dL)	2.9 µM (116 µg/dL)	2.7 µM (108 µg/dL)	2.0 µM (80 µg/dL)
Lathosterol	<5.0 µM (<200 µg/dL)	**5.3 µM (212 µg/dL)**	1.9 µM (76 µg/dL)	2.3 µM (92 µg/dL)	1.9 µM (76 µg/dL)	4.6 µM (184 µg/dL)	**6.0 µM (240 µg/dL)**	4.9 µM (196 µg/dL)
Lanosterol	<0.4 µM (16 µg/dL)	0 µM (0 µg/dL)	0.2 µM (8 µg/dL)	0 µM (0 µg/dL)	0.2 µM (8 µg/dL)	0.3 µM (12 µg/dL)	0 µM (0 µg/dL)	0.3 µM (12 µg/dL)
24-Dihydrolanosterol	0 µM (0 µg/dL)	0 µM (0 µg/dL)	0 µM (0 µg/dL)	0 µM (0 µg/dL)	0 µM (0 µg/dL)	0 µM (0 µg/dL)	0 µM (0 µg/dL)	0 µM (0 µg/dL)

Results out of the reference range are shown in bold. Values in parentheses are conventional units.

Abbreviations: µM, µmol/L; HDL-C, high-density lipoprotein cholesterol; LDL-C, low-density lipoprotein cholesterol; mM, mmol/L; Prava, pravastatin.

## Treatment

Both patients were already treated with ezetimibe in monotherapy at 10 mg daily when the diagnosis of sitosterolemia was confirmed. Following this diagnosis, ezetimibe was increased to 10 mg twice a day and a low-phytosterol diet was initiated. Both patients met with a registered nutritionist to learn how to follow a low-phytosterol diet. Patient 2 was also treated with pravastatin, which was associated with further improvement of her blood lipid profile (Fig. 1D).

## Outcome and Follow-up

Following the implementation of the low-phytosterol diet, a reduction of up to 27.6% and 22.4% of the plasmatic phytosterol levels was observed in patient 1 and patient 2, respectively (see Table 1). The platelet count and MVP both normalized after treatment with ezetimibe and the low-phytosterol diet (see Fig. 1). Following these treatment changes, they did not complain of any symptoms related to CVD or musculoskeletal diseases and did not report the appearance of new xanthomas or xanthelasmas.

## Discussion

Recent studies suggest that the estimated prevalence of sitosterolemia is around 1 in 200 000 individuals among the general population. This is a much higher frequency than previously reported (16). Nevertheless, this condition is thought to be substantially underdiagnosed and patients tend to be diagnosed late (2), as was the case for our 2 patients.

Sitosterolemia is probably underdiagnosed because a high clinical suspicion is needed to make the diagnosis. Clinically, sitosterolemia should be considered in patients with hypercholesterolemia, tendon xanthomas, and unexplained hemolysis or macrothrombocytopenia, as these hematologic abnormalities are not present in FH or in other monogenic causes of hypercholesterolemia. Hematologic abnormalities are often the initial presentation of sitosterolemia and can be the only clinical features of the disorder (16-18). Therefore, a complete blood count should be performed, and the patient's past medical history should be carefully reviewed to assess any unexplained hematologic anomalies. Platelet count and MPV are 2 important biomarkers of this rare disease and are useful to monitor the response to treatment. Indeed, our patients showed macrothrombocytopenia at the initial presentation and had normalization of their platelet counts and MPV after the initiation of the appropriate treatment.

The diagnosis of sitosterolemia is often made after an individual with severe hypercholesterolemia suspected as having FH fails to achieve expected low-density cholesterol (LDL-C) reduction with statin therapy or has negative FH-related genetic investigations (19). Multigene panels for FH often do not include *ABCG5* and *ABCG8* genes, and this could be another reason for the underdiagnosis of sitosterolemia (7). We deeply believe that these 2 genes should be included in FH multigene panels to permit an earlier distinction between the 2 disorders as the clinical management of sitosterolemia highly differs from other causes of hypercholesterolemia. Unfortunately, genetic testing can sometime give uncertain results, such as for the 2 VUS identified in 1 of our patients. Interpretation of VUS is often challenging, and the opinion of a medical geneticist can be helpful in the medical-decision process. In this case, the 2 VUS were not considered disease-causing, but we cannot rule out that these VUS could partially contribute to the patient's phenotype.

Obviously, routine lipid profiles do not distinguish plant sterols from cholesterol. Detection of elevated phytosterol concentrations in plasma requires conducting a sterol profile using gas-liquid chromatography, gas chromatography/mass spectrometry, or high-pressure liquid chromatography (7). Although identification of increased plasma phytosterols can lead to the diagnosis of sitosterolemia, a sterol profile may not be available or routinely requested during the initial diagnostic workup. We would highly recommend including a sterol profile in the diagnostic workup in atypical cases of FH as the identification of elevated phytosterols could be the first clue for the appropriate diagnosis (2, 3, 7).

Early diagnosis of sitosterolemia is very important as early treatment can be associated with better outcomes (7). Moreover, inappropriate diagnosis and treatments could lead to complications in patients. Indeed, the treatment options for patients with sitosterolemia differ considerably from those of other causes of hypercholesterolemia. The usual dietary recommendations of increasing intake of fruits, vegetables, nuts, and of using unsaturated fats from plants can be harmful to patients with sitosterolemia and contribute to the worsening of atherosclerotic CVD (3, 7). As noted, both of our patients had evidence of atherosclerotic CVD prior to their sitosterolemia diagnosis, and one patient had even demonstrated worsened CV symptoms following the initiation of a Mediterranean diet. This could probably have been avoided, or at least limited, if they had received the appropriate diagnosis earlier.

Dietary restriction of cholesterol and noncholesterol sterols is the nutritional intervention of choice for sitosterolemia patients (3, 7, 16). Unfortunately, clear nutritional recommendations for sitosterolemia are currently lacking. A plant sterol–free diet is very difficult to achieve for patients, and information about sitosterol content is lacking on product labeling. Moreover, dietary changes are often not sufficient to improve the sterol profile and pharmacological treatment is often required. Although statins could be useful to reduce LDL-C in these patients, they do not represent the best pharmacological option. Ezetimibe, which inhibits the gastrointestinal absorption of sterols, is the first-line drug therapy for sitosterolemia. It is associated with a 10% to 50% lowering in LDL-C levels, phytosterols, the stabilization of xanthomas, and the improvement of hematologic complications (19). Bile acid sequestrants can also be considered in those with an incomplete response to ezetimibe (20). Combination with an anti-PCSK9 agent has also been described in the literature (21). There is currently no clear consensus regarding target level of LDL-C or phytosterol in sitosterolemia (16). Ideally, prospective randomized controlled trials investigating whether specific lowering of LDL-C and phytosterol leads to reduced risk for CVD, as well as for other complications of sitosterolemia, should be performed.

In conclusion, this case review illustrates the importance of considering rare conditions, such as sitosterolemia, as a differential diagnosis in patients presenting with atypical hypercholesterolemia. Appropriate diagnosis is of the utmost importance as the treatment of sitosterolemia differs from other causes of hypercholesterolemia and the usual treatments recommended to individuals at higher risk of CVD could be harmful to these patients. Early diagnosis and treatment could lead to improved clinical outcomes and allow for appropriate genetic counseling.

## Learning Points

- Sitosterolemia is a rare autosomal recessive disorder often confused clinically with FH, and an accurate diagnosis is crucial as treatment highly differs between the two conditions.
- Sitosterolemia should be considered even in patients with probable FH, especially if they are presenting with macrothrombocytopenia and absent or reduced clinical response to statin.
- Sitosterolemia can be accurately diagnosed using a sterol profile and genetic testing.
- Early treatment with a low phytosterol diet and ezetimibe can improve symptoms and prognosis.
- Response to treatment can be followed by assessing platelet count and MPV in patients presenting with macrothrombocytopenia.

## Data Availability

Original data generated and analyzed for this case report are included in this published article.

## Abbreviations

CVD: cardiovascular disease
FH: familial hypercholesterolemia
LDL-C: low-density lipoprotein cholesterol
MVP: mean platelet volume
VUS: variants of uncertain significance
